# Reliability of a Wearable Motion System for Clinical Evaluation of Dynamic Lumbar Spine Function

**DOI:** 10.31031/acam.2022.07.000660

**Published:** 2022-07-06

**Authors:** Hamed Hani, Reid Souchereau, Anas Kachlan, Halle Harris, Jonathan Dufour, Alexander Aurand, Prasath Mageswaran, Madison Hyer, William Marras

**Affiliations:** 1 Spine Research Institute, The Ohio State University, USA; 2 Department of Integrated Systems Engineering, The Ohio State University, USA; 3 Center for Biostatistics, Department of Biomedical Informatics, The Ohio State University, USA

**Keywords:** Low back disorder, Reliability, Intra-rater, Inter-rater, Wearables, Lumbar function

## Abstract

**Background:**

Low back pain is the leading cause of disability worldwide. Subjective assessments are often used to assess extent of functional limitations and treatment response. However, these measures have poor sensitivity and are influenced by the patient’s perception of their condition. Currently, there are no objective tools to effectively assess the extent of an individual’s functional disability and inform clinical decision-making.

**Objective:**

The purpose of this study was to evaluate the reliability of a wearable motion system based on Inertial Measurement Unit (IMU) sensors for use in quantifying low back function.

**Methods:**

Low back motion assessments were conducted by 3 novice raters on 20 participants using an IMU-based motion system. These assessments were conducted over 3 days with 2 days of rest in between tests. A total of 37 kinematic parameters were extracted from the low back motion assessment in all three anatomical planes. Intra-rater and inter-rater reliability were assessed using Intraclass Correlation Coefficients (ICCs) calculated from repeated measures, mixed-effects regression models.

**Results:**

Lumbar spine-specific kinematic parameters showed moderate to excellent reliability across all kinematic parameters. The ICC values ranged between 0.84–0.93 for intra-rater reliability and 0.66 – 0.83 for inter-rater reliability. In particular, velocity measures showed higher reliabilities than other kinematic variables.

**Conclusion:**

The IMU-based wearable motion system is a valid and reliable tool to objectively assess low back function. This study demonstrated that lumbar spine-specific kinematic metrics have the potential to provide good, repeatable metrics to assess clinical function over time.

## Introduction

Among all Musculoskeletal Disorders (MSDs), Low Back Pain (LBP) is the leading cause of long- term disability and the most prevalent condition with a lifetime prevalence of 65–80% in USA adults [1]. LBP prevalence is on the rise, imposing a financial burden to employers and healthcare systems [2,3]. Identifying root causes of LBP has been a challenge for physicians and researchers, as LBP is associated with many different biopsychosocial factors [4]. Traditionally, practitioners have utilized subjective methods to quantify back pain, such as questionnaires or surveys [5–8]. While these methods may provide insights into perceptions of pain intensity, they do not represent objective measures that describe biomechanical or functional dimensions of LBP. Therefore, a simple and reliable clinical tool that holistically evaluates and provides an objective snapshot into the functional status of the lumbar spine may be helpful in stratifying LBP patients, monitoring treatment outcomes, and enhancing clinical decision-making to improve long-term outcomes. There have been several biomechanical tools developed to assess the functional state of a patient with LBP. Goniometers and digital inclinometers are frequently utilized for the assessment of lumbar range of motion [9–13]. The reliability of these goniometric measures assessed via Intraclass Correlation Coefficients (ICCs) range from 0.85–0.99, and >0.95 for digital inclinometry [11]. However, while these measurement tools provide reliable measures of flexibility, they only provide two-dimensional kinematic information under static conditions and do not necessarily capture the complex dynamics of functional human motion. Moreover, the sensitivity and specificity of lumbar range of motion to differentiate between LBP patients and healthy cohorts are both poor and questionable [14].

**Figure 1:**
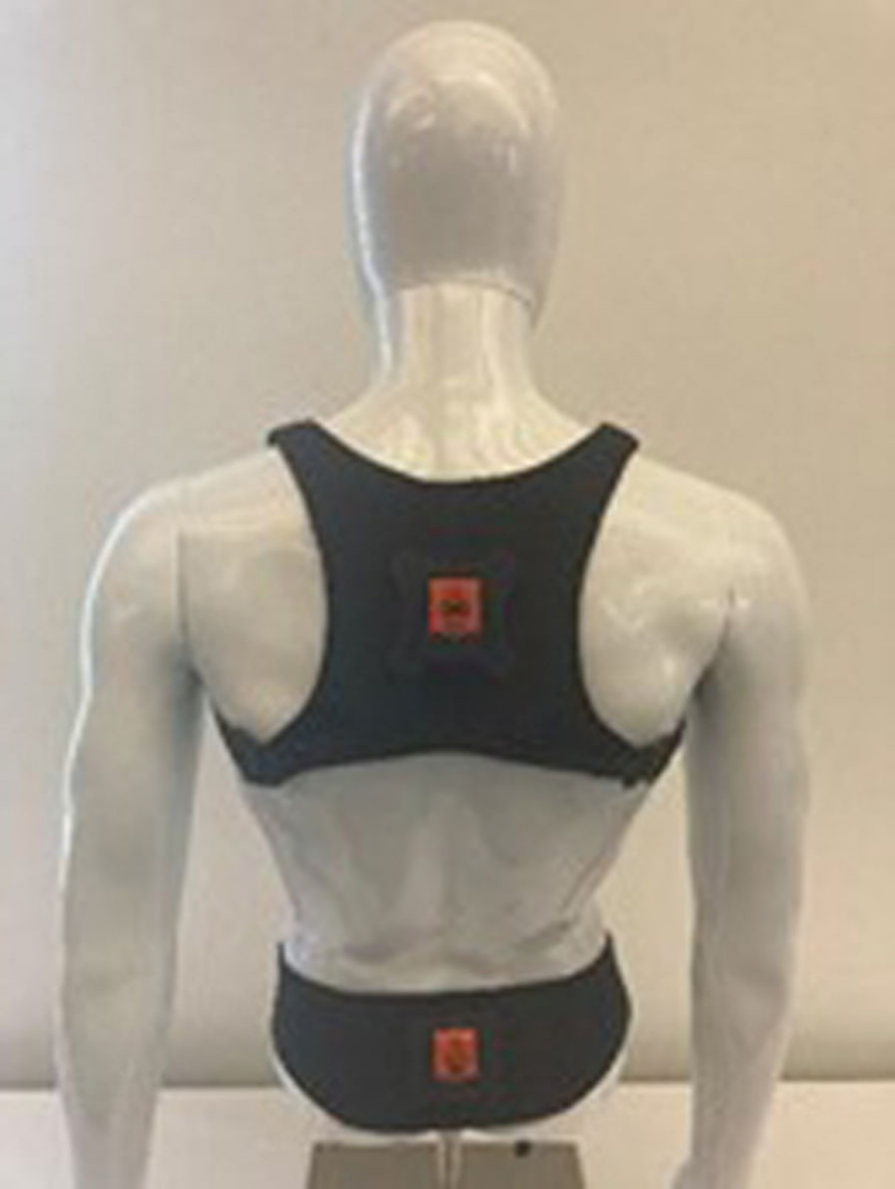
IMU sensor configuration for Lumbar Spine Motion Assessment

In contrast, Inertial Measurement Units (IMUs) are portable and cost-effective motion sensors that can capture three-dimensional dynamic movement data and have the potential to serve as an objective tool to evaluate the functional status of the spine [15]. The reliability and performance of IMUs compared to the gold standard optical motion capture system have been evaluated in prior literature with promising results. Beange et al. [16] recruited 10 subjects to perform 35 cycles of repetitive spine flexion-extension. The reliability of IMUs relative to an optical motion capture system for measuring features associated with spine movement and motor control showed strong ICC values ranging between 0.81–0.96. A similar study by Bauer et al. [17] also investigated the performance of an IMU system for use in the assessment of movement dysfunctions. In their study, the concurrent validity of the IMU system was compared to an optoelectronic system. Their results demonstrated that when compared to the optoelectronic system, the IMU system is valid for estimates of trunk movement in the primary movement direction. Finally, Kang et al. [18] examined the validity of IMUs when measuring the mean postural angles for thorax and head flexion, and shoulder girdle elevation during gait in 7 healthy individuals. For assessment of the accuracy, Bland-Altman analysis and ICC values (>0.73) showed promising results confirming the agreement of IMUs with the motion capture system. With the need for objective measures and the utility of IMUs to provide useful reliable measures, recently, the authors developed a novel IMU-based lumbar spine wearable device (Figure 1) for clinical use to provide a direct objective measure of functional spine health. While wearing the device, an individual performs a standardized 10-minute functional motion assessment. The device automatically captures the individual’s unique motion signature while they perform these series of standardized multi-planar, lumbar-specific functional motion tasks. The motion signature consists of three-dimensional (3D) lumbar range of motion (Room) as well as dynamic features such as velocities and accelerations. Collectively, these lumbar-specific motion signature data provide a window into the functioning of an individual’s musculoskeletal system and can serve as indicators of functional health. However, the reliability of this IMU-based functional motion assessment needs to be further investigated to better inform clinical utility and acceptance. Therefore, the purpose of this study was to quantify the reliability of lumbar kinematic features captured within an integrated software platform that interacts with an IMU-based lumbar spine wearable device. Inter- and intra- rater reliability were quantified with healthy controls to understand the total variance across various raters at different time points. The results of this study form a foundation for future work that will utilize the same or similar IMU-based platforms to quantify low back function.

## Material and Methods

### Subjects

A total of 20 healthy participants were evaluated in this study, consisting of 10 males and 10 females aged 18–64 years. Twenty participants were chosen as the number needed to determine statistical significance as it has been established by previous studies [19].

#### Inclusion criteria:

Participants that were eligible for this study include those who aged between 18 and 80 years old. Participants must have been able to stand for 25 minutes at a time and able to speak, read, and understand English so that instructions and safety precautions were understood.

#### Exclusion criteria:

Our exclusion criteria were participants with back pain or history of chronic low back pain, history of any severe brain or spine pathologies, uncorrected vision impairment, uncorrected hearing impairment, vestibular conditions requiring medical treatment, bone fractures within the last 3 months, and chemotherapy or radiation therapy in the last 3 months.

### Clinical lumbar motion assessment

#### Software and hardware components:

A unique cloud-based software application was leveraged for all data collection, storage, review, filtering, sorting, and processing operations. The functional lumbar spine motion assessment was performed using commercially available IMU sensors (XSens MTw2) integrated into custom lightweight flexible harnesses. The wearable device was designed specifically for collecting three-dimensional lumbar-specific kinematics non-invasively. The system was designed for safe human contact, with the sensors mounted on harnesses worn over clothing. All hardware and software components allow for the accurate recording of kinematic variables with built-in sensor drift correction algorithms [20].

#### Study design

A repeated measures experimental design using three raters was used to assess intra-rater and inter-rater reliability. The raters were inexperienced first-time users of the technology. As part of the study protocol, they were briefly trained on the use of the technology prior to subject enrollment and data collection. Each participant was evaluated independently by the three raters on three separate days (9 total data collection evaluations). A 30-minute rest period was provided to each participant between motion tests by rater on the same day. In addition, two days of rest were also required between each day to minimize the impact of learning and fatigue effects onparticipants. Rater order was counterbalanced across days and participants. Raters were blind to tests performed by other raters. A typical participant schedule is shown in Table 1.

### Detailed procedures

Prior to enrollment, the study details were explained, and each participant signed a consent form. Sufficient time was given to participants to review their consent forms and ask additional questions. Upon enrollment into the study, each participant completed a series of questionnaires that captured information on their current pain, fatigue, and low back stiffness. On completion of these questionnaires, the participants then performed a 10-minute functional motion assessment using a wearable IMU-based system for the motion assessment, lightweight harnesses (belt and vest) with mounted IMU sensors were placed on the upper torso and pelvis of participants as shown in Figure 1. Then, each participant was asked to perform a series of pre-defined low back motions in each of three anatomical planes (lateral, axial, and sagittal). The lateral motion trials had subjects lean their upper body to the left and right without moving their pelvis. The axial motion trials had subjects rotate their upper body to the left and right while avoiding pelvis movement. The sagittal motion trials had the subject flex their upper back forward and then return to the starting position. The software provided verbal instructions for each trial and asked participants to perform the motions as fast or as far as they could comfortably. The software also permitted practice for each motion task and provided advanced instructions if the tasks were not performed properly. The first three motion trials captured multi-planar range of motion (flexibility), and the latter five trials captured dynamic motions (see Supplemental Section for details on motion assessment). These motions were directly recorded to the custom software application. The participant completed the study after performing a total of 9 data collection sessions.

### Statistical analysis

Low back motion signals were characterized into a series of features including range of motion and mean or peak velocities and mean or peak accelerations. Details on these features have been outlined and described in Section 10 in the appendix. Intraclass Correlation Coefficients (ICCs) were calculated to investigate intra-rater and inter-rater reliability of low back motion assessments using repeated measures, mixed-effects regression models. All ICCs [ICC (2,1) and ICC (3,1)] were calculated and reported in accordance with Koo and Li [21]. Based on the guidelines set by Koo and Li [21], ICC values less than 0.5 indicate poor reliability, values between 0.5 and 0.75 indicate moderate reliability, values between 0.75 and 0.9 indicate good reliability, and values above 0.9 indicate excellent reliability. All analyses were performed using SAS v9.4 and R. The type I error rate was alpha = 0.05.s

## Results

### Subject characteristics

Table 2 displays the baseline demographic characteristics of the subjects (participants). Subjects comprised of 10 males and 10 females with age ranges of 18–64 years. In total, 70% (n=14) were white, 25% (n=5) were Asian, and 5% (n=1) were unknown. In addition, the mean height and weight of these subjects were 171.5±9.6 cm (67.5±3.8 inches) and 69.8±11.9 kg (153.8±26.2 pounds), respectively.

### Intra-rater reliability

Overall, we analyzed the reliability of 37 kinematic measures captured during functional motion. Table 3 displays the intra-rater ICC estimates and 95% confidence intervals as a function of each of the cardinal planes for the various motion tasks. The intra-rater reliability for mean flexibility (RoM) in the axial plane was moderate to excellent with a mean ICC=0.85 (95% CI: 0.56–0.96), whereas mean axial velocity and acceleration showed higher intra-rater reliabilities with ICC=0.89 (95% CI: 0.80–1.00) and ICC=0.87 (95% CI: 0.80–0.94), respectively. For the lateral plane, all motion metrics (flexibility, velocity, and acceleration) yielded reliability estimates ranging from good to excellent. More specifically, ICC estimates for mean lateral flexibility, velocity, and accelerations were 0.91 (95% CI: 0.84–0.94), 0.92 (95% CI: 0.86–0.96), and 0.87 (95% CI: 0.78–0.95), respectively. In addition, all intra-rater reliability values for the sagittal plane of the body resulting from the sagittal symmetric task, sagittal bending while twisted Clockwise (CW) task, and sagittal bending while twisted Counterclockwise (CCW) task yielded good to excellent results with mean ICC estimates ranging between 0.86–0.93 for flexibility, velocity, and acceleration metrics. Collectively, the intra-rater ICC estimates suggest good to excellent reliability for all metrics of velocity and acceleration as well as flexibility except in the axial plane of the body.

### Inter-rater eliability

The inter-rater reliability findings (Table 4) resulted in a slightly different pattern compared with intra-rater reliability. That is, this study found that mean axial flexibility resulted in poor to good inter-rater reliability with ICC=0.69 (95% CI: 0.29–0.87), whereas mean axial velocity showed moderate to excellent reliability with ICC=0.77 (95% CI: 0.62–1.00). The mean axial acceleration metric also resulted in moderate to excellent reliability with ICC=0.73 (95% CI:0.60–1.00). With regards to the lateral plane, kinematic metrics produced inter-rater reliability estimates that were moderate to good with mean ICC estimates ranging between 0.60–0.80. For the sagittal symmetric tasks that yielded sagittal plane flexion and extension flexibility and velocity metrics, ICC values ranged between 0.76–0.80. The flexion and extension sagittal acceleration measures also yielded reliability in the moderate range (ICC values ranging 0.71–0.72), whereas mean sagittal acceleration yielded inter-rater reliability from moderate to excellent with ICC= 0.74 (95% CI: 0.60–1.00). For the sagittal asymmetric tasks that yielded sagittal velocity and acceleration metrics when flexing and extending while twisting in a counterclockwise position, the reliability metrics ranged from moderate too good for all velocity and acceleration measures (ICC values ranging 0.75–0.83). Finally, sagittal velocity and acceleration metrics when flexing and extending while twisting in a clockwise position resulted in good reliability for all velocity metrics and moderate reliability for all acceleration metrics. While performing the task twisted clockwise, extension sagittal velocity showed the best reliability with ICC=0.77 (95% CI: 0.63–0.86) in velocity metrics and mean sagittal acceleration showed the best reliability with ICC=0.75 (95% CI: 0.59–1.00) in acceleration metrics.

### Reliability patterns among metrics

**Figure 2:**
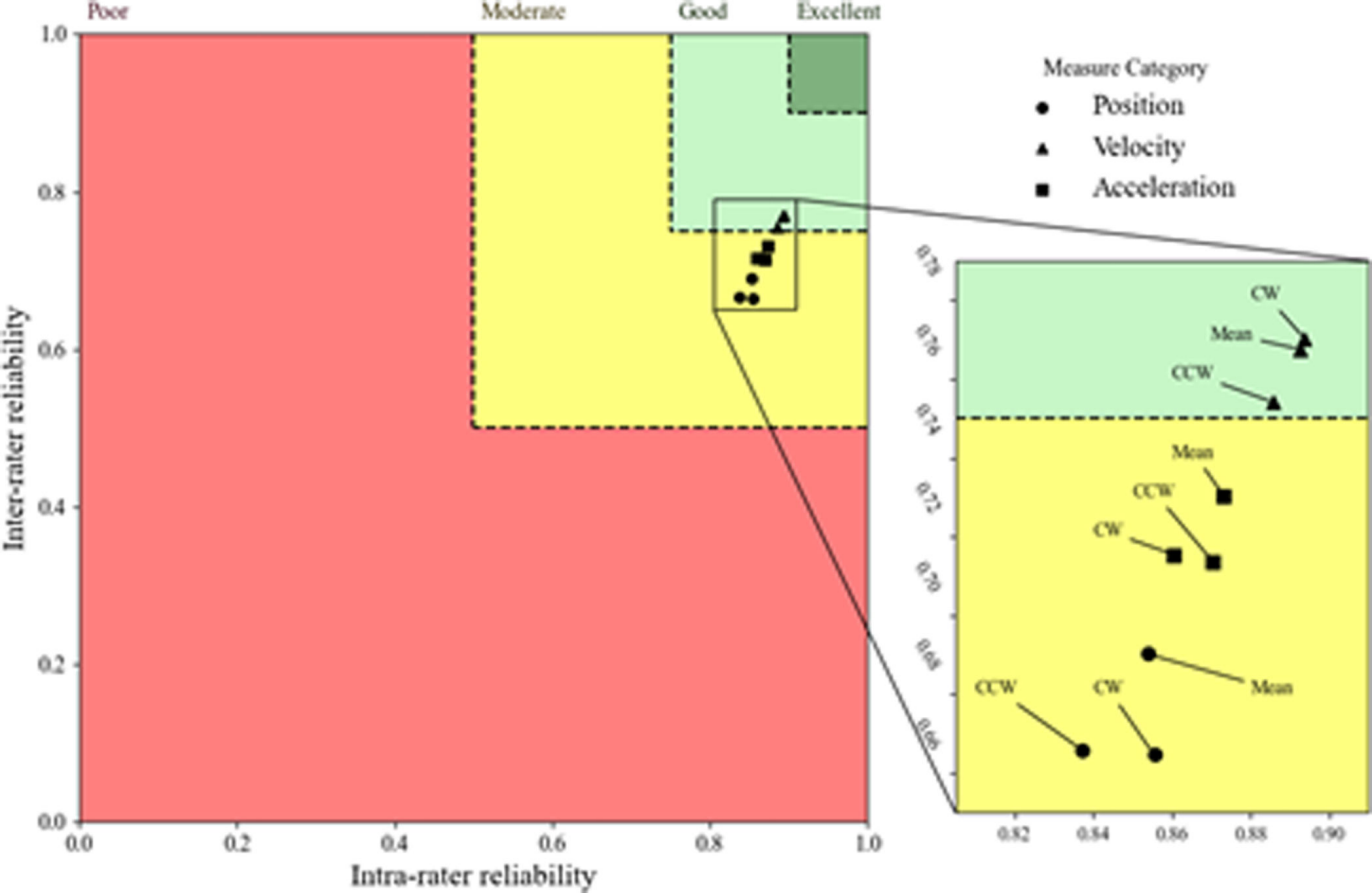
Inter- versus intra-rater reliability in the axial plane. Poor, moderate, good, and excellent reliability regions are displayed in red, yellow, light green, and dark green colors, respectively. Positional, velocity-related, and acceleration-related measures are shown with circles, triangles, and squares.

**Figure 3:**
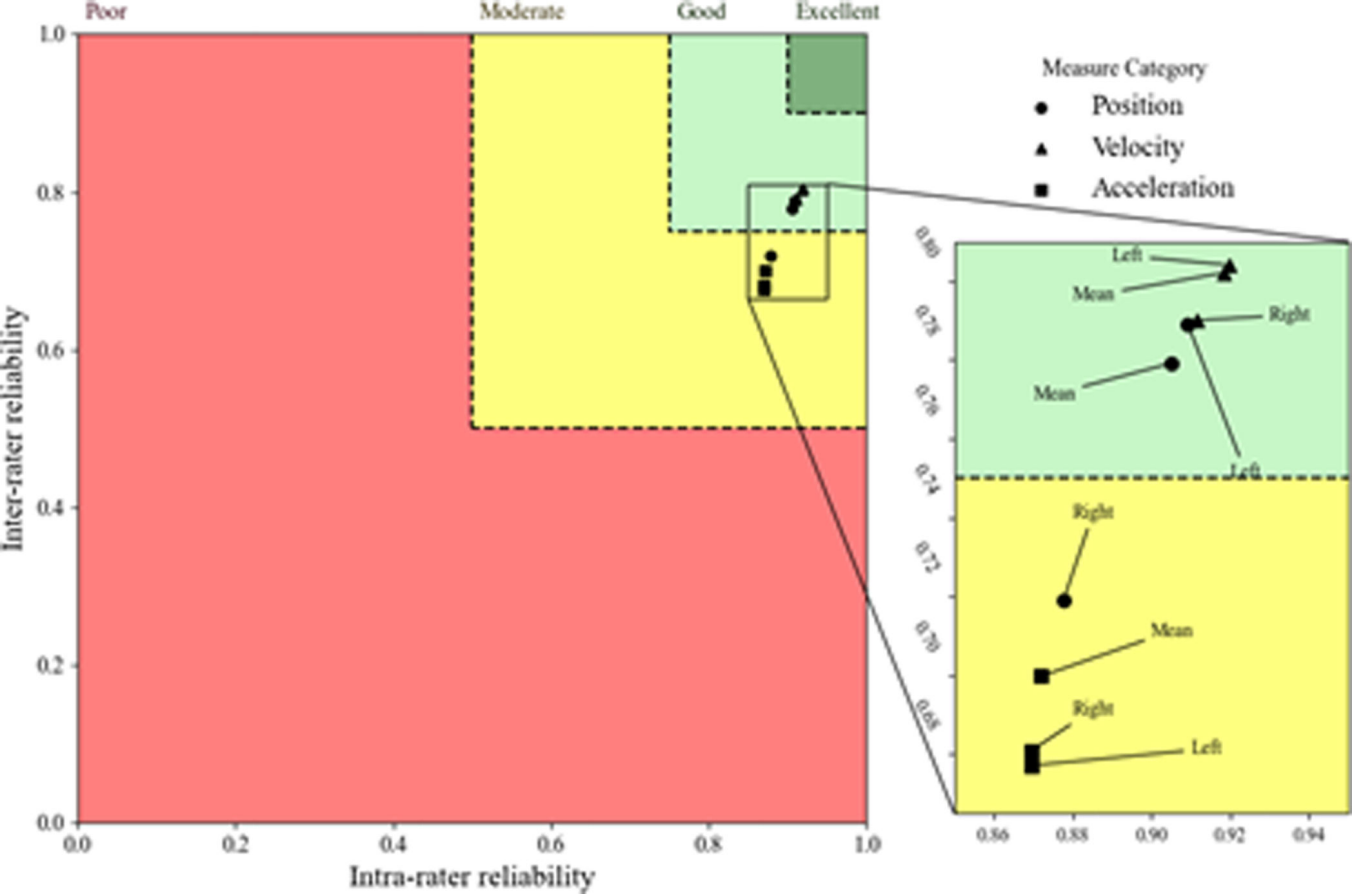
Inter- versus intra-rater reliability in the lateral plane. Poor, moderate, good, and excellent reliability regions are displayed in red, yellow, light green, and dark green colors, respectively. Positional, velocity-related, and acceleration-related measures are shown with circles, triangles, and squares.

**Figure 4:**
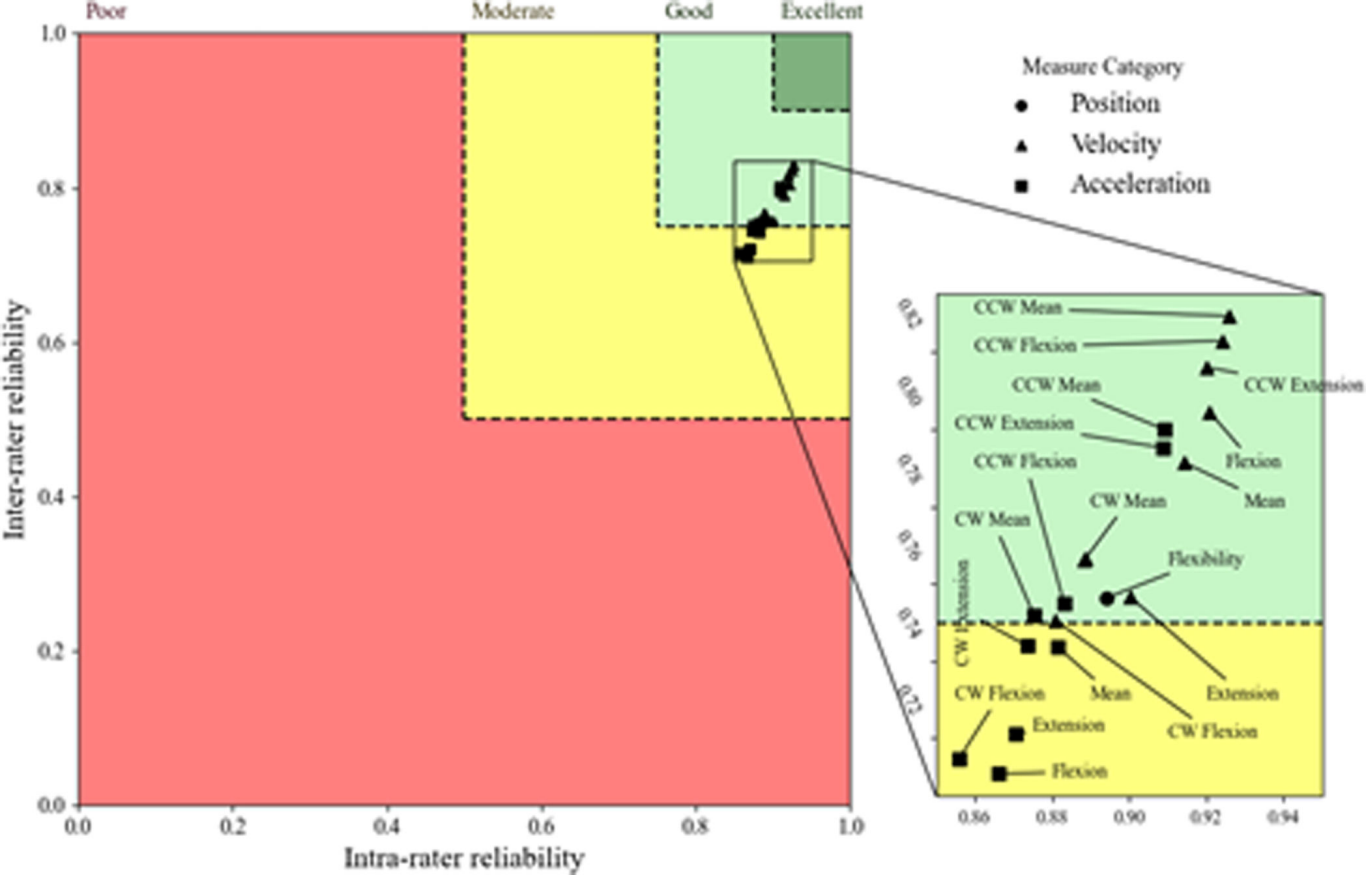
Inter- versus intra-rater reliability in the sagittal plane. Poor, moderate, good, and excellent reliability regions are displayed in red, yellow, light green, and dark green colors, respectively. Positional, velocity-related, and acceleration-related measures are shown with circles, triangles, and squares.

**Figure 5:**
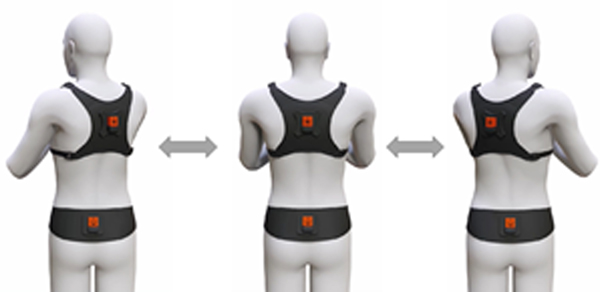

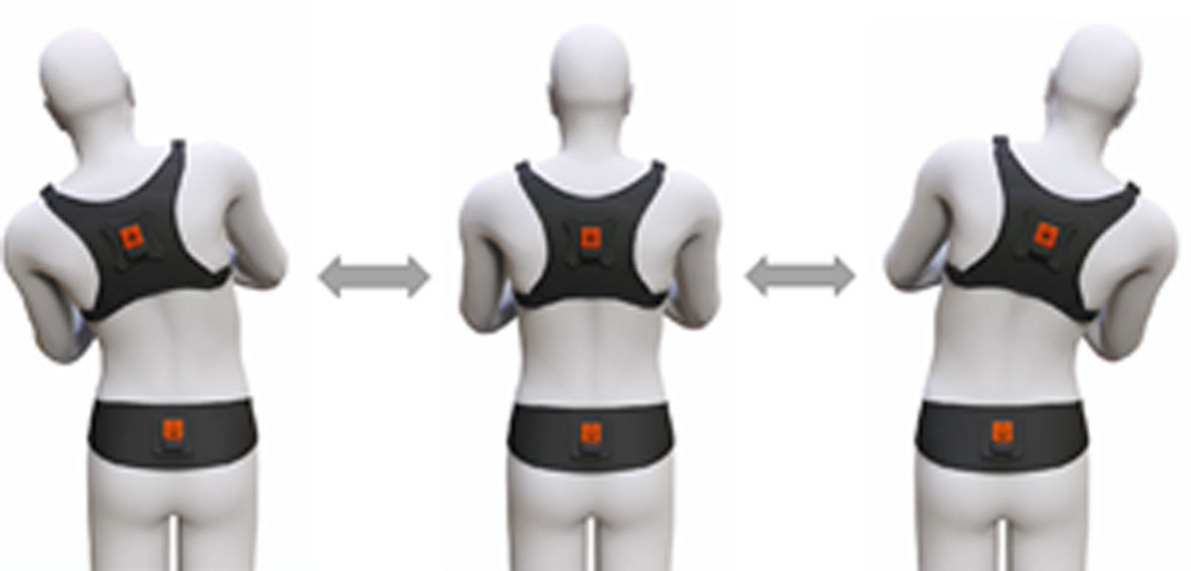

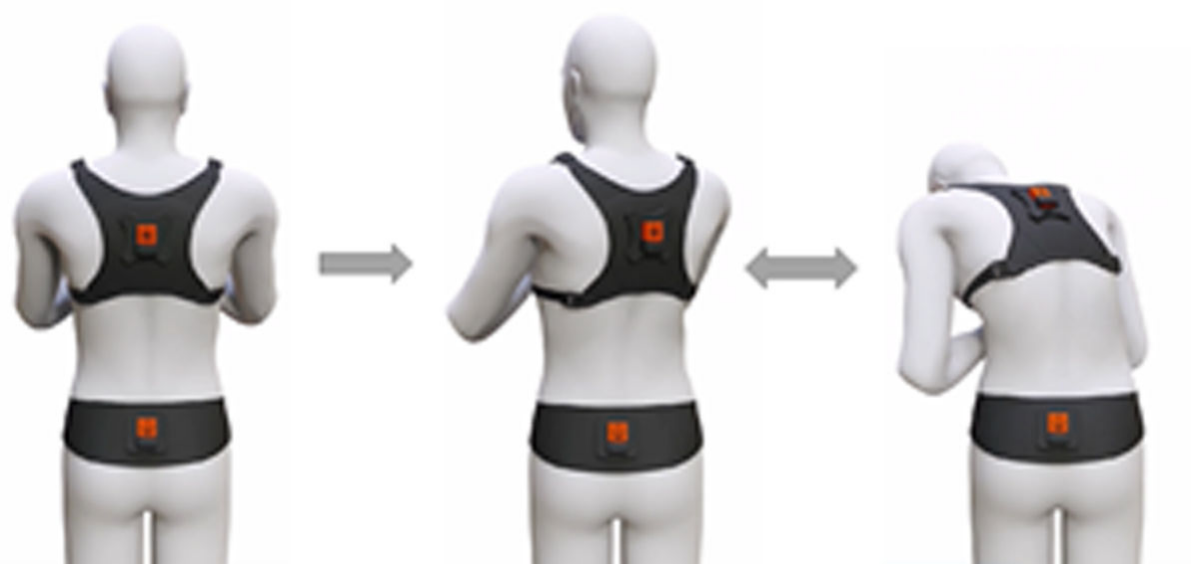

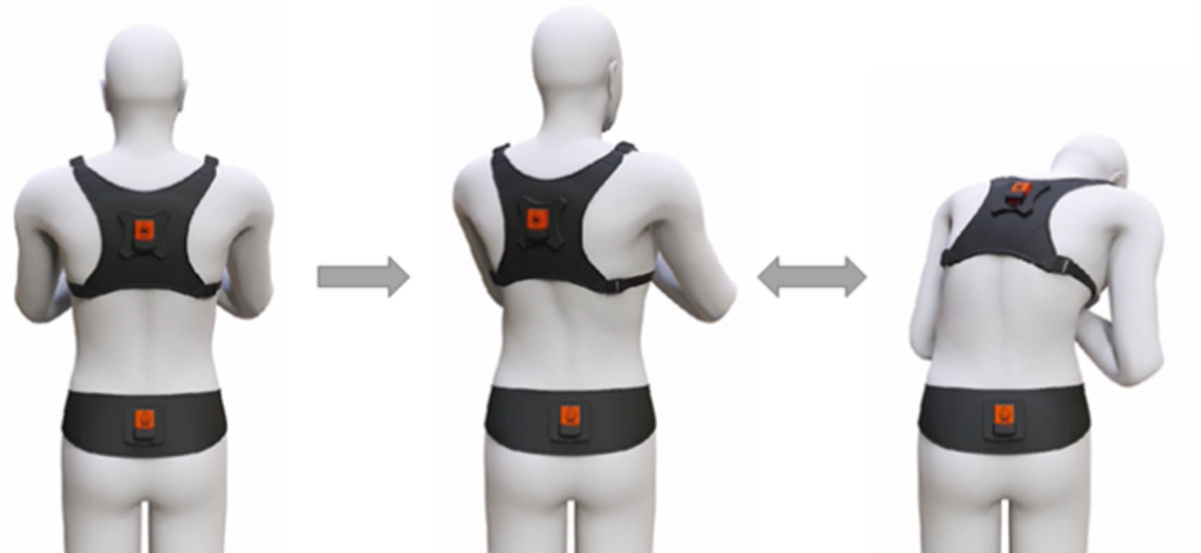

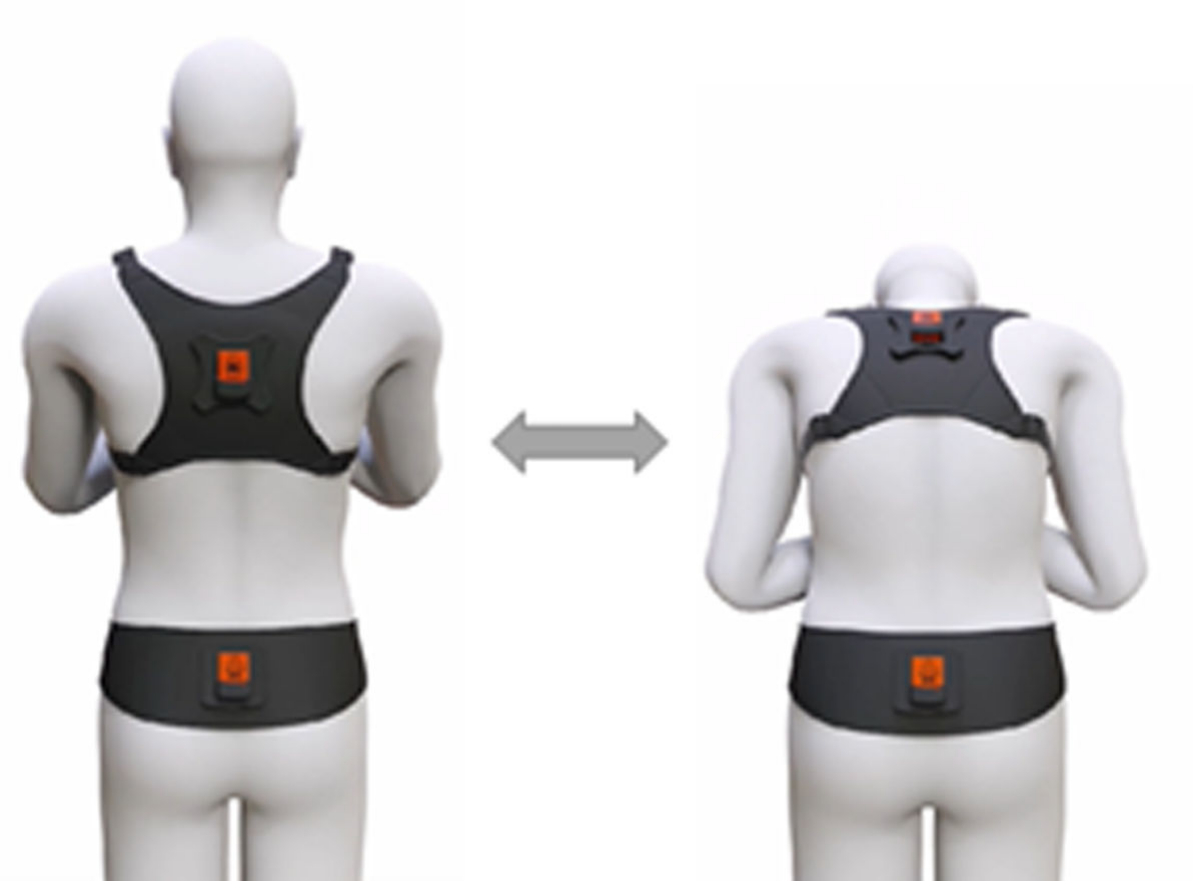
Illustration of performing each motion.

When considering the inter-rater and intra-rater results together, an interesting pattern in the ICC distributions was evident. More specifically, velocity measures were distributed towards higher ICCs for all tasks compared with both flexibility and acceleration metrics in all planes. (Figures 2–4) demonstrate ICCs of the various kinematic metrics relative to the inter-rater vs intra- rater reliability for the axial, lateral, and sagittal planes, respectively. These plots were intended to indicate the estimates of the ICC distributions so that one can appreciate the relative relationship among the various kinematic metrics. In these figures, poor, moderate, good, and excellent reliability regions are displayed in red, yellow, light green, and dark green colors, respectively, so that the relationships between the intra-rater and inter-rater ICC mean estimates could be considered collectively. Positional measures are shown with circles, measures related to velocity are shown with triangles, and measures related to acceleration are shown with squares. These figures indicate that the mean ICC estimates for all kinematic metrics were in the good or excellent range in all anatomical planes. There were two main trends that can be noted from these plots. First, the inter-rater and intra-rater ICCs within the sagittal and lateral planes of the body produced ICC estimates that were generally better than those for the axial plane of the body. Second, in all planes of the body, ICCs were better for the velocity kinematic metrics than for the other kinematic metrics in nearly every instance (Figure 5).

## Discussion

**Figure 6:**
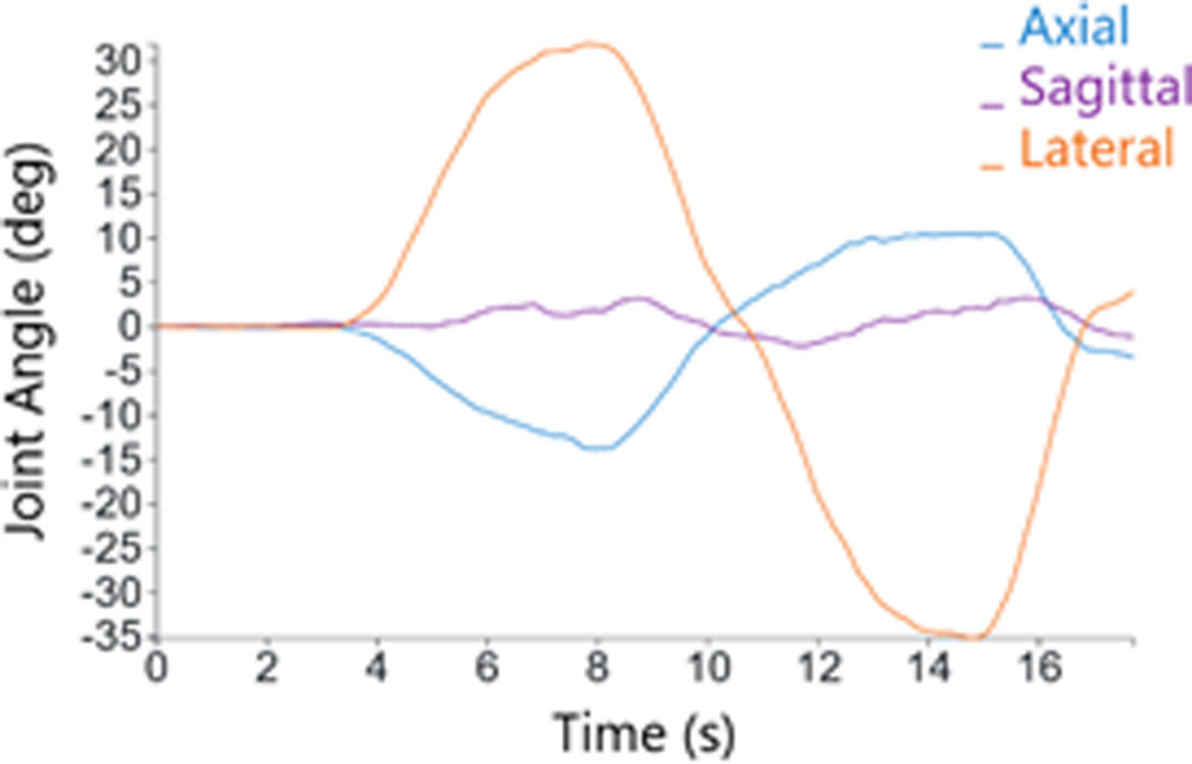

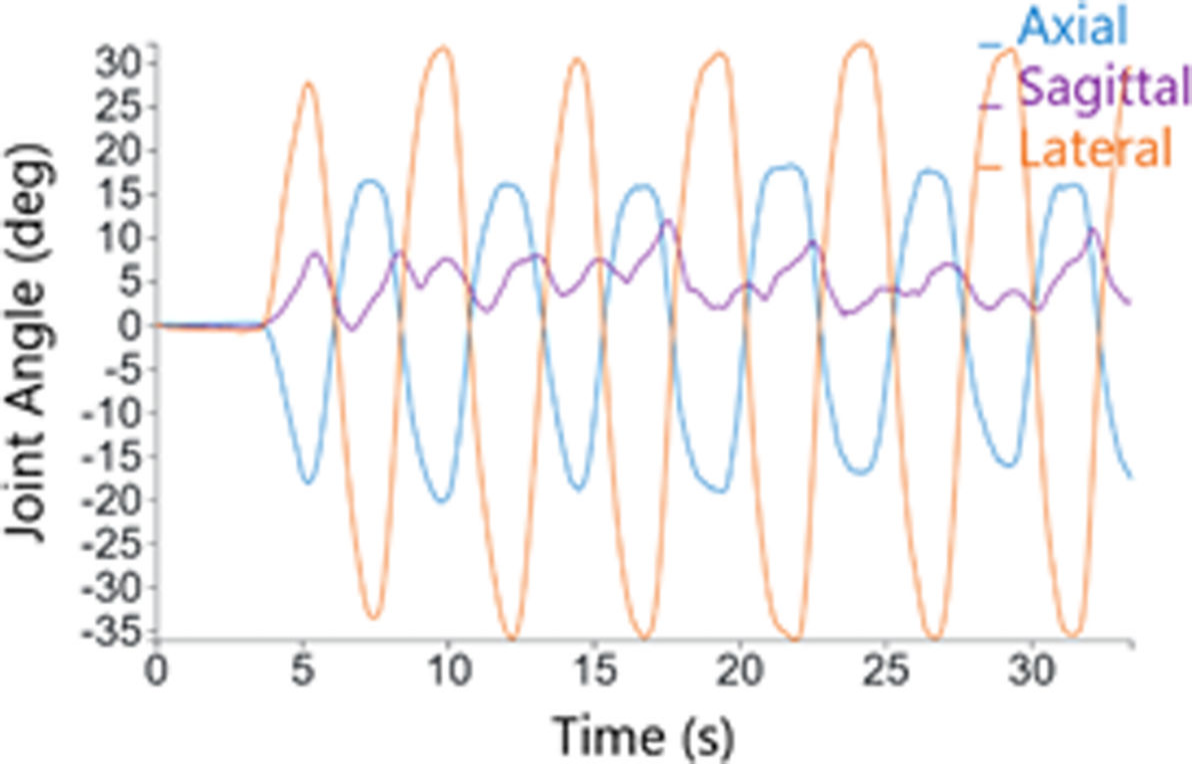
Lateral motion data collected with device.

The current study evaluated the reliability of an IMU-based wearable motion system to provide quantitative kinematic metrics indicative of low back function. Our results showed the extent to which three-dimensional lumbar kinematics were reliable and can serve as useful functional indicators of spine health. Overall, the ICC values for the wearable motion system showed good to excellent intra-rater and moderate to good inter-rater reliabilities. Two dimensions of intra-rater and inter-rater reliability were assessed in this study (Figure 6). The results of this study demonstrated that there are many spines kinematic metrics derivable from the various functional motion tasks that yield “moderate” to “excellent” and “moderate” to “good” reliability. Of particular note between the intra-rater and inter-rater test reliability results is the idea that the velocity-related metrics resulted in ICC values that were generally greater than other kinematic parameters like ROM or acceleration metrics. It is particularly noteworthy that the intra-rater reliability was particularly good in the lateral and sagittal planes. This information should be especially useful when deriving unique composite metrics of functional spine status that combine some of these metrics. It should also be noted that the wearable system used here was extremely accurate in terms of measuring spine motion. The system has been compared to a high-fidelity infra-red motion capture system and found to be within 98.1%, 99.5%, and 99.0% accuracy of the infra-red camera system when measuring position, velocity, and acceleration, respectively [20]. Thus, these reliability measures reflected the repeatability of the test due to variation between raters and variation by the subjects over time.

The ICC estimates in our study compared favorably (and in many cases slightly better) to other studies that have reported ICCs for the torso and other parts of the body. Marras et al. [14] investigated repeatability of trunk kinematic metrics using a wearable lumbar motion monitor in 20 subjects repeating the same tasks over the course of five weeks with one rater. Their study reported Cronbach’s alpha correlation coefficients as a reliability measure for various kinematic measures in the sagittal and lateral planes of the body. As in the current study, Marras et al found very good repeatability for velocity and acceleration in the sagittal plane and moderate repeatability in the lateral plane of the body. Simmonds et al. [22] assessed test-retest reliability of physical performance using a series of trunk flexion movements with one rater and 44 low back patients and 48 healthy subjects. Day-to-day reliability (ICC) reported values ranged between 0.59–0.88 in the low back pain group and between 0.46–0.76 in the control group. The ICCs reported in our current study were generally better than these. Another study investigated trunk strength repeatability as opposed to kinematics examined in the current study. Rabelo et al. [23] investigated inter-rater and test-retest reliability of trunk strength and endurance measures from an isokinetic dynamometer. In this study, 13 patients and 13 healthy subjects were recruited and performed a series of trunk flexion and extension movements using different movement protocols. The reported ICC values ranged between 0.59 – 0.99. This further suggests that the vast majority of the kinematic metrics assessed via our IMU-based wearable system were generally more reliable, especially considering the lower bounds of the ICCs reported by Rabelo et al. [23]. Unlike the current study, other studies have reported better repeatability for position of the trunk. O’Sullivan et al. [24] found reliable ICCs when monitoring trunk position. In their study, they evaluated the reliability of a wireless spinal position monitoring device on 18 healthy participants while they performed sitting and forward bending tasks. They reported between-day ICC values from 0.84–0.87 and inter-rater ICC values ranging from 0.91–0.94. Similarly, Intolo et al. [25] investigated the validity and reliability of a Spineangel^®^ device on 18 healthy males and reported excellent ICC values for trunk range of motion (ICC>0.90).

The ICCs reported in our study were slightly higher for intra-rater compared to inter-rater reliability. This implies that there was a slightly higher degree of agreement between days of testing (time points) compared to the agreement between raters. In addition, variability of measures might also be due to the subjects varying day to day rather than the raters or testing method itself. Subjects did not report significant changes in stiffness or pain; thus, it is possible that uncontrolled external factors, which might include sleep, nutrition, exercise outside of the study, or psychosocial fluctuations, impacted the subjects’ movement. It is also possible that a learning effect occurred between sessions (Table 5). More in-depth analyses suggested slightly worse agreement in metrics on the first day of testing compared to subsequent test days. We suspect that subjects became familiar with the motions after completing them multiple times. Despite these limiting factors, the findings of good to excellent ICCs for motion metrics within intra-rater reliability and moderate to good ICCs for motion metrics with inter-rater reliability indicates relative consistency between and within raters. Hence, overall, these results reflect a reliable set of kinematic metrics that objectively reflect low back function. There were several limitations to this study. As mentioned previously, some external factors were not measured that might have impacted motion assessments, such as sleep, nutrition, exercise, or psychosocial factors. Some of these effects might be mitigated through the study design and calculations of reliability because motion assessments administered on the same day would likely not exhibit large variations of these factors. Another potential limitation was that the motion signals were not explored for more complex features, outside of traditional kinematic measures, that could prove to be reliable metrics. However, even with these potential limitations, this study demonstrates that many of the metrics reported herein can serve as reliable indicators of low back function and has the potential to provide useful clinical information.

## Conclusion

The lumbar spine measurement system discussed herein has demonstrated good reliability for both inter-rater and intra-rater reliability via ICCs. For each measure, the intra-rater reliability was slightly greater than the inter-rater reliability. The intra-rater reliability ranged from moderate to excellent among the various kinematic metrics with most of these measures indicating good to excellent reliability. Similarly, inter-rater reliability ranged from moderate to good with the exception of some of the axial flexibility plane metrics, which revealed poor to good repeatability. Both velocity and acceleration measures in the different anatomical planes had higher reliability scores than the flexibility measures. These reliability results suggest that these lumbar spine-specific kinematic metrics have a high reliability for evaluating low back function for different raters on the same day. The metrics also displayed some variability between days that were most likely a result of subtle subject changes between days. Given this information, adjustments to testing protocols might further improve the measurement (Table 6). However, overall, this study has demonstrated that kinematic metrics as measured by this IMU-based wearable device can provide very good, repeatable metrics to assess the functional low back status of individuals over time.

## Figures and Tables

**Table 1.** Experiment design

Day 1			Day 2			Day 3		
1	2	3	4	5	6	7	8	9
Test 1	Test 2	Test 3	Test 1	Test 2	Test 3	Test 1	Test 2	Test 3
Rater A	Rater B	Rater C	Rater B	Rater C	Rater A	Rater C	Rater A	Rater B

**Table 2.** Highlights subject characteristics

Characteristic	Mean (stdev) or n (%)
***Age***	
18 – 20	1 (5%)
20 – 25	8 (40%)
25 – 30	2 (10%)
30 – 35	3 (15%)
35 – 40	1 (5%)
40 – 45	-
45 – 50	-
50 – 55	2 (10%)
55 – 60	1 (5%)
Over 60	2 (10%)
***Gender***	
Male	10 (50%)
Female	10 (50%)
***Race***	
White	14 (70%)
Asian	5 (25%)
Unknown	1 (5%)
***Height & Weight***	
Height (cm)	171.5 (9.6)
Weight (kg)	69.8 (11.9)

**Table 3.** Intra-rater reliability estimates for all measures

Measure	ICC	2.50%	97.50%
CCW Axial Flexibility	0.84	0.57	0.95
CW Axial Flexibility	0.86	0.61	0.94
Mean Axial Flexibility	0.85	0.56	0.96
CCW Axial Velocity	0.89	0.79	1.00
CW Axial Velocity	0.89	0.80	1.00
Mean Axial Velocity	0.89	0.80	1.00
CCW Axial Acceleration	0.87	0.79	0.95
CW Axial Acceleration	0.86	0.79	0.92
Mean Axial Acceleration	0.87	0.80	0.94
Left Lateral Flexibility	0.91	0.84	0.95
Right Lateral Flexibility	0.88	0.80	0.93
Mean Lateral Flexibility	0.91	0.84	0.94
Left Lateral Velocity	0.92	0.86	0.96
Right Lateral Velocity	0.91	0.84	0.96
Mean Lateral Velocity	0.92	0.86	0.96
Left Lateral Acceleration	0.87	0.77	1.00
Right Lateral Acceleration	0.87	0.77	1.00
Mean Lateral Acceleration	0.87	0.78	0.95
Flexion Sagittal Flexibility	0.89	0.80	0.95
Extension Sagittal Velocity	0.9	0.82	0.95
Flexion Sagittal Velocity	0.92	0.85	0.96
Mean Sagittal Velocity	0.91	0.84	0.96
Extension Sagittal Acceleration	0.87	0.75	0.96
Flexion Sagittal Acceleration	0.87	0.77	0.94
Mean Sagittal Acceleration	0.88	0.79	0.96
CCW Sagittal Extension Velocity	0.92	0.85	0.96
CCW Sagittal Flexion Velocity	0.92	0.86	0.96
CCW Sagittal Mean Velocity	0.93	0.86	0.96
CCW Sagittal Extension Acceleration	0.91	0.80	1.00
CCW Sagittal Flexion Acceleration	0.88	0.81	0.94
CCW Sagittal Mean Acceleration	0.91	0.82	1.00
CW Sagittal Extension Velocity	0.89	0.80	0.94
CW Sagittal Flexion Velocity	0.88	0.78	0.94
CW Sagittal Mean Velocity	0.89	0.79	0.94
CW Sagittal Extension Acceleration	0.87	0.75	0.96
CW Sagittal Flexion Acceleration	0.86	0.77	0.92
CW Sagittal Mean Acceleration	0.88	0.77	0.95

**Table 4.** Inter-rater reliability estimates for all measures

Measure	ICC	2.50%	97.50%
CCW Axial Flexibility	0.67	0.32	0.86
CW Axial Flexibility	0.66	0.32	0.84
Mean Axial Flexibility	0.69	0.29	0.87
CCW Axial Velocity	0.75	0.60	1.00
CW Axial Velocity	0.77	0.61	1.00
Mean Axial Velocity	0.77	0.62	1.00
CCW Axial Acceleration	0.71	0.56	0.88
CW Axial Acceleration	0.72	0.60	0.82
Mean Axial Acceleration	0.73	0.60	1.00
Left Lateral Flexibility	0.79	0.67	0.87
Right Lateral Flexibility	0.72	0.57	0.81
Mean Lateral Flexibility	0.78	0.66	0.86
Left Lateral Velocity	0.80	0.70	0.88
Right Lateral Velocity	0.79	0.67	0.88
Mean Lateral Velocity	0.80	0.69	0.89
Left Lateral Acceleration	0.68	0.54	0.79
Right Lateral Acceleration	0.68	0.53	0.81
Mean Lateral Acceleration	0.70	0.56	0.82
Flexion Sagittal Flexibility	0.76	0.58	0.86
Extension Sagittal Velocity	0.76	0.62	0.86
Flexion Sagittal Velocity	0.80	0.69	0.89
Mean Sagittal Velocity	0.79	0.66	0.88
Extension Sagittal Acceleration	0.72	0.56	0.87
Flexion Sagittal Acceleration	0.71	0.57	0.84
Mean Sagittal Acceleration	0.74	0.60	1.00
CCW Sagittal Extension Velocity	0.82	0.68	0.90
CCW Sagittal Flexion Velocity	0.82	0.70	0.91
CCW Sagittal Mean Velocity	0.83	0.71	0.91
CCW Sagittal Extension Acceleration	0.80	0.62	1.00
CCW Sagittal Flexion Acceleration	0.75	0.63	1.00
CCW Sagittal Mean Acceleration	0.80	0.65	1.00
CW Sagittal Extension Velocity	0.77	0.63	0.86
CW Sagittal Flexion Velocity	0.75	0.59	0.86
CW Sagittal Mean Velocity	0.77	0.62	0.86
CW Sagittal Extension Acceleration	0.74	0.56	1.00
CW Sagittal Flexion Acceleration	0.71	0.59	0.83
CW Sagittal Mean Acceleration	0.75	0.59	1.00

**Table 5.** Glossary of movements

Term	Interpretation
Axial movement (Figure 5.a)	Rotating the upper body in a plane that separates the body upper and lower halves as much as possible to the CCW or CW
Lateral movement (Figure 5.b)	Rotating the upper body in a plane that separates the body front and back halves as much as possible to the CCW or CW
CCW sagittal movement (Figure 5.c)	Rotating the upper body in a plane that separates the body upper and lower halves as much as possible to the CCW and performing the sagittal movement
CW sagittal movement (Figure 5.d)	Rotating the upper body in a plane that separates the body upper and lower halves as much as possible to the CW and performing the sagittal movement
Sagittal movement (Figure 5.e)	Tilting the upper body in a plane that separates the body CCW and CW halves as much as possible forward
Flexibility trial (Figure 6.a)	A trial which any rotational motion is performed for one cycle in a controlled manner as far as comfortably possible to achieve extreme rotational position
Motion trial (Figure 6.b)	A trial which any rotational motion is performed multiple cycles as fast as comfortably possible to achieve extreme rotational speed and acceleration

**Table 6.** Glossary of kinematic variables

Measure	Description
CCW Axial Flexibility	How far counterclockwise (CCW) the subject twists in the axial plane
CW Axial Flexibility	How far clockwise (CW) the subject twists in the axial plane
Mean Axial Flexibility	The mean absolute (regardless of direction) flexibility in the axial plane collectively in both CCW and CW directions
CCW Axial Velocity	Peak velocity the subject twists CCW in the axial plane
CW Axial Velocity	Peak velocity the subject twists CW in the axial plane
Mean Axial Velocity	The mean absolute (regardless of direction) peak velocity in the axial plane both the CCW and CW directions
CCW Axial Acceleration	Peak acceleration while twisting CCW in the axial plane
CW Axial Acceleration	Peak acceleration while twisting CW in the axial plane
Mean Axial Acceleration	Mean peak absolute (regardless of direction) acceleration in the axial plane in the CCW and CW directions
Left Lateral Flexibility	How far to the subject’s left the subject bends in the lateral plane
Right Lateral Flexibility	How far right to the subject’s the subject bends in the lateral plane
Mean Lateral Flexibility	The mean (regardless of direction) flexibility in the lateral plane in both the left and right directions
Left Lateral Velocity	The peak velocity while a subject bends to their left side in the lateral plane
Right Lateral Velocity	The peak velocity while a subject bends to their right side in the lateral plane
Mean Lateral Velocity	The mean peak absolute (regardless of direction) velocity in the lateral plane while a subject bends in both the left and right sides
Left Lateral Acceleration	The peak acceleration while the subject bends to their left in the lateral plane
Right Lateral Acceleration	The peak acceleration while the subject bends to the right side of the lateral plane
Mean Lateral Acceleration	The mean absolute (regardless of direction) peak acceleration in the lateral plane in both the left and right sides
Flexion Sagittal Flexibility	How far forward the subject can bend in the sagittal plane
Extension Sagittal Velocity	The peak Velocity as the subject bends back to neutral from a flexed position in the sagittal plane
Flexion Sagittal Velocity	The peak Velocity as thesubject bends forward in the sagittal plane
Mean Sagittal Velocity	The mean peak velocity when the subject bends forward and back to neutral in the sagittal plane
Extension Sagittal Acceleration	The peak acceleration as the subject bends back to neutral from flexion in the sagittal plane
Flexion Sagittal Acceleration	The peak acceleration as the subject bends forward in the sagittal plane
Mean Sagittal Acceleration	The mean absolute (regardless of direction) peak acceleration when a subject bends forward and back to neutral in the sagittal plane
CCW Sagittal Extension Velocity	The peak velocity as subject bends back to neutral from a flexed position in the sagittal plane while fully twisted CCW
CCW Sagittal Flexion Velocity	The peak velocity as the subject bends forward in the sagittal plane from a neutral position while fully twisted CCW
CCW Sagittal Mean Velocity	The mean peak velocity as subject bends forward and back to neutral in the sagittal plane while fully twisted CCW
CCW Sagittal Extension Acceleration	The peak acceleration a subject has while bending back to neutral in the sagittal plane when fully twisted CCW
CCW Sagittal Flexion Acceleration	The peak acceleration as the subject bends forward in the sagittal plane when fully twisted CCW
CCW Sagittal Mean Acceleration	The mean peak acceleration as the subject bends forward and back to neutral in the sagittal plane while fully twisted CCW
CW Sagittal Extension Velocity	The peak velocity as the subject bends back to neutral in the sagittal plane while fully twisted CW
CW Sagittal Flexion Velocity	The peak velocity as the subject bends forward in the sagittal plane while fully twisted CW
CW Sagittal Mean Velocity	The peak velocity as the subject bends forward and back to neutral in the sagittal plane while fully twisted CW
CW Sagittal Extension Acceleration	The peak acceleration as the subject bends back to neutral in the sagittal plane while fully twisted CW
CW Sagittal Flexion Acceleration	The peak accelerationas the subject bends forward in the sagittal plane while fully twisted CW
CW Sagittal Mean Acceleration	The peak absolute (regardless of direction) acceleration as the subject bendsforward and back to neutral in the sagittal plane while fully twisted CW

## References

[1] Urits I, Burshtein A, Sharma M, Lauren T, Peter A, (2019) Low back pain, a comprehensive review: pathophysiology, diagnosis, and treatment. Curr Pain Headache Rep 23(3): 23. 30854609 10.1007/s11916-019-0757-1

[2] Dutmer AL, Schiphorst PHR, Soer R, Sandra B, Ute B, (2019) Personal and societal impact of low back pain. Spine (Phila Pa 1976) 44(24): E1443–E1451. 31369481 10.1097/BRS.0000000000003174

[3] Wu A, March L, Zheng X, Jinfeng H, Xiangyang W, (2020) Global low back pain prevalence and years lived with disability from 1990 to 2017: Estimates from the global burden of disease study 2017. Ann Transl Med 8(6): 299. 32355743 10.21037/atm.2020.02.175 PMC7186678

[4] Alhowimel A, AlOtaibi M, Radford K, Coulson N (2018) Psychosocial factors associated with change in pain and disability outcomes in chronic low back pain patients treated by physiotherapist: A systematic review. SAGE Open Med 6: 2050312118757387. 10.1177/2050312118757387 PMC5808969 29449945

[5] Mannion AF, Balagué F, Pellisé F, Cedraschi C (2007) Pain measurement in patients with low back pain. Nat Clin Pract Rheumatol 3(11): 610–618. 17968331 10.1038/ncprheum0646

[6] Chiarotto A, Boers M, Deyo RA, Rachelle B, Terry P, (2018) Core outcome measurement instruments for clinical trials in nonspecific low back pain. Pain 159(3): 481–495. 29194127 10.1097/j.pain.0000000000001117 PMC5828378

[7] Carvalho FA, Morelhão PK, Franco MR, Chris G, Rob J, (2017) Reliability and validity of two multidimensional self-reported physical activity questionnaires in people with chronic low back pain. Musculoskelet Sci Pract 27: 65–70. 28637603 10.1016/j.msksp.2016.12.014

[8] Campbell P, Foster NE, Thomas E, Dunn KM (2013) Prognostic Indicators of low back pain in primary care: Five-Year Prospective Study. J Pain 14(8): 873–883. 23791041 10.1016/j.jpain.2013.03.013 PMC3739005

[9] Triano JJ, Schultz AB (1987) Correlation of objective measure of trunk motion and muscle function with low-back disability ratings. Spine (Phila Pa 1976) 12(6): 561–565. 2958944 10.1097/00007632-198707000-00010

[10] Romero FN, Jiménez RP, Montaño MJA (2017) Validity and reliability of a low-cost digital dynamometer for measuring isometric strength of lower limb. J Sports Sci 35(22): 2179–2184. 27882825 10.1080/02640414.2016.1260152

[11] Kolber MJ, Hanney WJ (2012) The reliability and concurrent validity of shoulder mobility measurements using a digital inclinometer and goniometer: a technical report. Int J Sports Phys Ther 7(3): 306–313. PMC3362980 22666645

[12] Gogia PP, Braatz JH, Rose SJ, Norton BJ (1987) Reliability and validity of goniometric measurements at the knee. Phys Ther 67(2): 192–195. 3809242 10.1093/ptj/67.2.192

[13] Youdas JW, Bogard CL, Suman VJ (1993) Reliability of goniometric measurements and visual estimates of ankle joint active range of motion obtained in a clinical setting. Arch Phy Med Rehabil 74(10): 1113–1118. 10.1016/0003-9993(93)90071-h 8215866

